# Evaluating the Accuracy of Laryngoscopic View Documentation During Tracheal Intubation in a Pediatric Emergency Department

**DOI:** 10.1016/j.acepjo.2025.100227

**Published:** 2025-07-24

**Authors:** Preston Dean, Maria Hooker, Benjamin T. Kerrey, Mary Frey, Yin Zhang, Stephanie Boyd, Karen Ahaus, Katherine Edmunds

**Affiliations:** 1 Division of Emergency Medicine, Cincinnati Children’s Hospital Medical Center, Cincinnati, OH, USA; 2 Department of Pediatrics, University of Cincinnati College of Medicine, Cincinnati, OH, USA; 3 Cincinnati Children’s Hospital Medical Center Residency Training Program, Cincinnati Children’s Hospital Medical Center, Cincinnati, OH, USA; 4 Division of Biostatistics and Epidemiology, Cincinnati Children’s Hospital Medical Center, Cincinnati, OH, USA

**Keywords:** airway, pediatrics, tracheal intubation

## Abstract

**Objectives:**

Review of self-reported data in the electronic medical record (EMR) is the standard approach to the study of emergency airway management. Despite this, very little research has been done into the accuracy of the laryngoscopic views documented in the EMR during intubation in the emergency department. Complicating matters further, the original Cormack-Lehane (CL) airway grading system and the newer modified CL grading system have overlapping definitions. The objective of this study was to compare the laryngoscopic views documented in the EMR to the laryngoscopic views seen on intraoral video during intubation in a pediatric emergency department.

**Methods:**

This was a retrospective review of prospectively collected observational data of patients undergoing intubation in an academic pediatric emergency department from February 2022 to February 2025. The main outcome was modified CL grade documented in the EMR vs modified CL grade seen on intraoral video. Correctly graded airways were defined as the modified CL grade documented in the EMR matching the best modified CL grade seen on intraoral video.

**Results:**

Airways were often incorrectly graded, occurring in 68 out of 161 attempts (42.2%). The most common CL grade discrepancy was documentation of a grade 1 view in the EMR when the best view on video was grade 2a or 2b (n = 63).

**Conclusion:**

Future airway research must account for potential inaccuracies in self-reported laryngoscopic views and/or inconsistencies between the use of the original versus modified CL airway grading system. More research is needed into which aspects of airway management are inaccurately recorded in the EMR.

**The Bottom Line**

Little research has been done into the accuracy of laryngoscopic views documented in the electronic medical record (EMR) during emergency tracheal intubation, and the original Cormack-Lehane (CL) airway grading system and modified CL grading system have overlapping definitions. Thus, we performed an observational study in an academic pediatric emergency department designed to compare laryngoscopic views documented in the EMR to laryngoscopic views seen on intraoral video. Airways were often incorrectly graded, occurring in 68/161 attempts (42.2%). Future research must account for potential inaccuracies in self-reported laryngoscopic views and/or inconsistencies between the use of the original versus modified CL grading system.

## 1 Introduction

### 1.1 Background

Multiple studies have found that intubation documentation in the electronic medical record (EMR) is often incomplete.^1^, ^2^, ^3^ Quality improvement initiatives have attempted to improve the completeness of intubation documentation in the EMR^1^, ^2^, ^3^; however, there has been little investigation into the accuracy of intubation documentation.

### 1.2 Importance

If intubation documentation is not accurate, we will be limited in our understanding of the true benefits and risks of evolving airway technology in children, including video laryngoscopy. Studies have shown that video-based intubation data collection produces higher-quality intubation data.^4^

### 1.3 Goals of This Investigation

The objective of this study was to compare laryngoscopic views documented in the EMR to laryngoscopic views on intraoral video laryngoscope recordings during intubations in a pediatric emergency department (PED).

## 2 Methods

### 2.1 Study Design

This was a retrospective review of prospectively collected observational data of patients undergoing intubation in an academic PED. This study was deemed exempt by our local institutional review board.

### 2.2 Setting

A video laryngoscope equipped with standard geometry blades (Storz C-MAC, Karl Storz) is used for >95% of intubations in our PED. Our resuscitation bays are equipped with video cameras and microphones. A four-view feed (2 overhead video recordings, patient vitals monitor, and the C-MAC video) can be reviewed using a proprietary software program (LiveCapture, B-Line Medical).

At our base PED, we typically perform 100 to 125 intubations per year, with pediatric emergency medicine (PEM) fellows performing >half of intubation attempts. The next most common groups to perform intubation, in descending order, are anesthesiologists, PEM faculty, and emergency medicine residents. Proceduralists typically document tracheal intubation using a procedure note in our EMR. The procedure note includes preprocedural details (preprocedural evidence of an anatomically difficult airway, limited mouth opening, cervical immobilization, medications used, etc), procedural details (number of attempts, airway grade, equipment used, etc), and postprocedural details (method of successful intubation confirmation, adverse events, depth of tube placement, etc). For airway grade, the proceduralist has the option to click 1 of 4 buttons (I, II, III, or IV, without additional definitions provided) or type in the airway grade via free text. The EMR does not specify which airway grading system the proceduralist should use or if the airway grade being documented is the best view, the most sustained view, or the view at the time of tube passage.

We enrolled a convenience sample of consecutive eligible patients who presented from February 2022 to February 2025.

### 2.3 Selection of Participants

Inclusion criteria were (1) patients intubated in our PED, (2) video recordings available, (3) EMR with a procedure note containing laryngoscopic views, and (4) intubation by a PEM physician (fellow or faculty) or emergency medicine resident. We included these proceduralists as they are the most reliable groups at writing procedure notes in our PED. We excluded patients who (1) were intubated with a direct laryngoscope, (2) were intubated during cardiopulmonary resuscitation, or (3) underwent multiple intubation attempts (as only 1 procedure note, and thus 1 laryngoscopic view, is documented).

### 2.4 Measurements

The main outcome was modified Cormack-Lehane (CL) grade. We compared the CL grade for the same patient attempt in the EMR versus intraoral video review. For comparison, we defined a correct CL grade as the grade in the EMR matching the best CL grade on video review. We also recorded the percentage of glottic opening (POGO) from the video.^5^^,^^6^

For the CL grade based on intraoral video review, videos were paused at the time the best view was obtained and the airway was independently graded by 2 study team members (PD and MH). One study team member (PD), with an extensive background in reviewing videos of emergency intubations including grading airway views, collected data at first opportunity after an airway case, usually within a few days after the attempt. A second study team member (MH) independently graded each available saved airway video recording. Both study team members were blinded to the other’s airway grade, as well as the grade documented in the EMR.

### 2.5 Analysis

We generated descriptive statistics for patient and proceduralist data and the outcomes of interest. Data are presented as presented as proportion (%) or median (IQR). To assess interrater reliability, we used weighted Cohen’s kappa.

## 3 Results

During the study period, 322 patients underwent intubation in our PED. After applying inclusion and exclusion criteria, our study sample was 161 patients (Figure S1). There was a high degree of interrater reliability between study team members who collected data (weighted Cohen’s kappa 0.854, 95% CI, 0.775-0.934).

Among the 161 intubations, laryngoscopic views of 134 (83.2%) intubations were documented in the EMR as grade 1. A total of 25 (15.5%) were documented as grade 2, and 1 (0.6%) was documented as grades 3 and 4. On intraoral video review, the best view obtained was grade 1 in 76/161 (47.2%) intubations and grade 2 in 81/161 (50.3%) intubations, and in no intubations was the best view obtained grade 3 or 4 (Table).

**Table:** Demographics and outcomes. Data are reported as median (IQR) or n (%).

Characteristic	Correctly graded, n = 93	Incorrectly graded, n = 68	Difference (95% CI)
Age (mo)	68.3 (18.4, 135.8)	70.2 (17.9, 148.5)	−1.5 (−40.2, 37.3)
Proceduralist			
First year PEM fellow	31 (33.3%)	30 (44.1%)	−10.8% (−26.0%, 4.4%)
Second year PEM fellow	22 (23.7%)	13 (19.1%)	4.5% (−8.2%, 17.3%)
PEM faculty^a^	23 (24.7%)	16 (23.5%)	1.2% (−12.2%, 14.6%)
EM resident	17 (18.3%)	9 (13.2%)	5.0% (−6.2%, 16.3%)
Documented CL grade			
1	71 (76.3%)	63 (92.6%)	−16.3% (−26.9%, −5.7%)
2	22 (23.7%)	3 (4.4%)	19.2% (9.3%, 29.2%)
3	0	1 (1.5%)	−1.5% (−4.3%, 1.4%)
4	0	1 (1.5%)	−1.5% (−4.3%, 1.4%)
Intraoral video view grade			
1	71 (76.3%)	5 (7.4%)	69.0% (58.4%, 79.6%)
2a	21 (22.6%)	60 (88.2%)	−65.7% (−77.1%, −54.2%)
2b	1 (1.1%)	3 (4.4%)	−3.3% (−8.6%, 2.0%)
3	0	0	--
4	0	0	--
Actual POGO	100 (100,100)	70 (55, 85)	30 (15, 45)
Blade type			
Macintosh	76 (81.7%)	59 (86.8%)	−5.0% (−16.3%, 6.2%)
Miller	16 (17.2%)	9 (13.2%)	4.0% (−7.2%, 15.1%)
D-blade	1 (1.1%)	0	1.1% (−1.0%, 3.2%)
Blade tip location			
Vallecula	78 (83.9%)	65 (95.6%)	11.7% (2.8%, 20.6%)
Epiglottis	15 (16.1%)	3 (4.4%)	−11.7% (−20.6%, −2.8%)
VL screen viewed	89 (95.7%)	60 (88.2%)	7.5% (−1.2%, 16.2%)

CL, Cormack-Lehane; EM, emergency medicine; IQR, interquartile range; PEM, pediatric emergency medicine; POGO, percentage of glottic opening; VL, video laryngoscope.

a Third year fellows considered as faculty, as this is the role they serve in our department.

Compared with video, the laryngoscopic view documented in the EMR was incorrectly graded in 68/161 (42.2%) of intubations (Table). The most common CL grade discrepancy was documentation of a grade 1 view in the EMR when the best view on video was grade 2a or 2b (n = 63). The median POGO for incorrectly graded intubations was 70 (IQR 55, 85), including examples of little to no glottic opening visualized despite being documented grade 1 (Figure).

**Figure:**
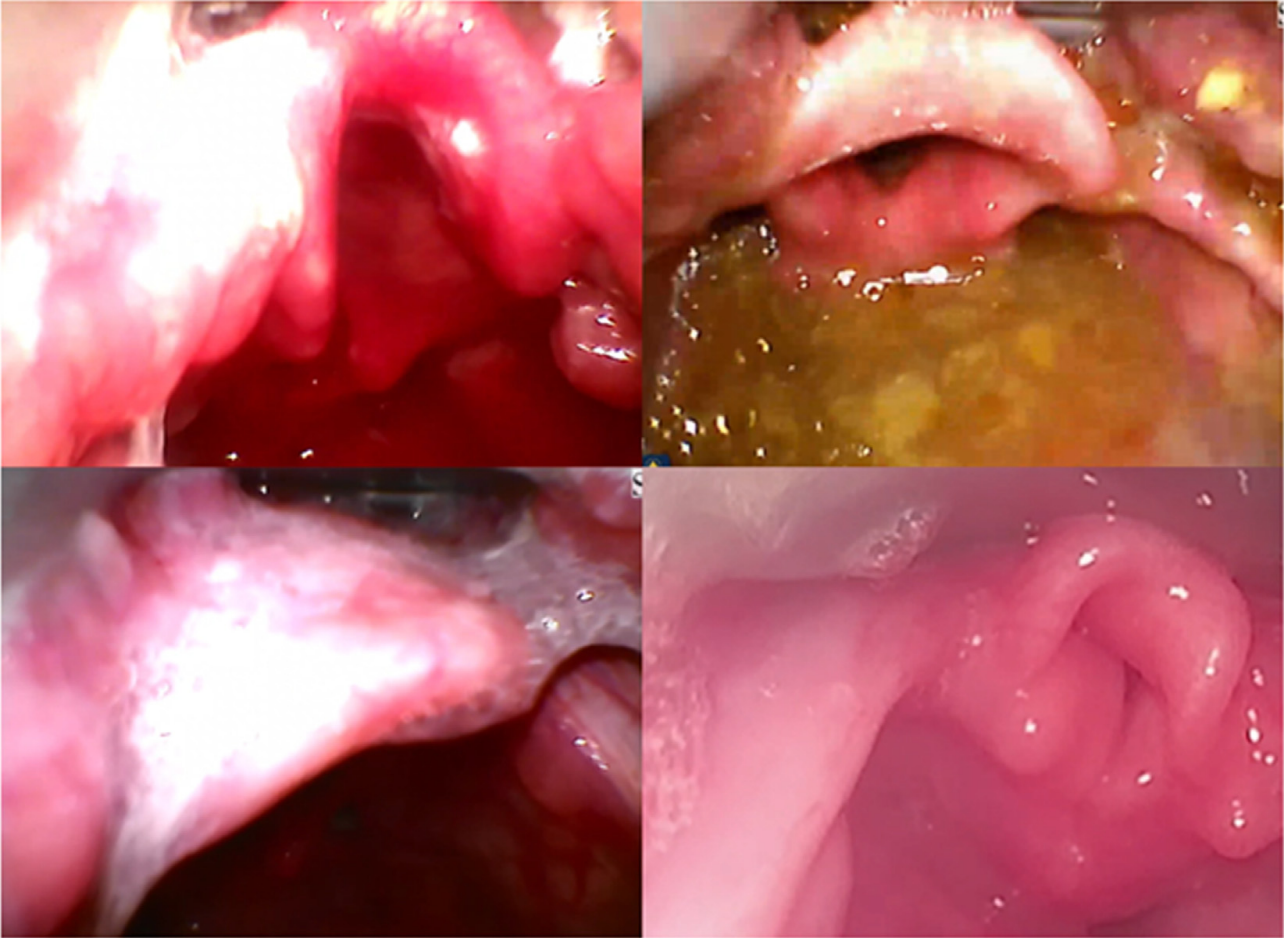
Example of best views obtained during intubations documented as grade 1.

Comparing correctly and incorrectly graded intubations, there was no difference in patient age (months), proceduralist type, blade type (Macintosh or Miller), or viewing of the video laryngoscope screen. The frequency of placing the laryngoscope tip in the vallecula (versus directly lifting the epiglottis) was higher in incorrectly graded intubations (Table).

## 4 Discussion

Our results should be interpreted in the context of several limitations. First, this was a single-center study in a PED where intubation is highly standardized. In particular, we have a 45-second limit on each intubation attempt, which may have caused proceduralists to attempt tube delivery before the optimal view was achieved. This possible early transition to tube delivery may have exaggerated the discrepancy seen between EMR documentation of airway grade and the views seen on intraoral videos had the time limit not been in place.

Second, because we use standard geometry blades, proceduralists had the ability to look at the video screen or directly into the patient’s mouth and could have obtained a better view by directly looking into the patient’s mouth. Because the screen was viewed in the vast majority of attempts, screen views are typically equal to or superior to direct views, and there was no difference in the frequency of viewing the video laryngoscope screen between correctly and incorrectly graded intubations; we do not believe this had any meaningful impact on our results.

Finally, and importantly, the original CL grading system and modified CL grading system have overlapping definitions, and it is possible these overlapping definitions could have contributed to the inaccurate interpretation of proceduralists’ airway grade documentation. Specifically, the original CL grading system, published in 1984, describes grade 1 as most of the glottis visible, whereas the modified CL grading system, published in 1998, describes grade 1 as the entire glottis visible.^6^^,^^7^ Although an important limitation, the overwhelming majority of proceduralists in this study completed training well after publication of the modified CL system and use of the terms “2a” and “2b” are common in our clinical environment, suggesting that most proceduralists in this study conceptualize airway grades using the modified CL system—although this is not certain. We also found many inexplicable discrepancies which would not be explained by the overlap of definitions or differences in interpretation, as 25% of incorrectly graded airways had POGO values of ≤50%, making them incorrectly graded in either the original or modified CL grading system.

Our findings suggest that documentation in the EMR based on self-report may often be inaccurate for some aspects of emergency airway management. Deliberate education on laryngoscopic view grading should be incorporated into training programs, and it should be clear whether laryngoscopic views are being graded using the original or modified CL grading system. Additionally, in the unique context of emergency intubations, describing the ease with which the view was obtained (did the patient require adjunct maneuvers, repositioning to improve airway alignment or significant force to achieve optimal view), as well as number of attempts and peri-intubation complications may be more important to document than CL grading.

More importantly, given that retrospective review of self-reported data in the EMR is the standard approach to data collection in resuscitation research, the implications of major discrepancies between the EMR and video are potentially wide-ranging. Future airway research must account for the potential for substantial inaccuracy of self-reported laryngoscopic views documented in the EMR, and future research endeavors are necessary to better understand factors that lead to incorrectly graded airways, as misrepresentation of an easy airway grade may lead to inadequate resources at the bedside.

## Author Contributions

PD, MH, BK, MF, YZ, SB, KA, and KE conceived and designed the study. PD performed the data collection. YZ provided the statistical advice and analyzed the data. PD drafted the manuscript, and all authors contributed substantially to its revision. PD takes full responsibility for the paper as a whole.

## Funding and Support

By *JACEP Open* policy, all authors are required to disclose any and all commercial, financial, and other relationships in any way related to the subject of this article as per ICMJE conflict of interest guidelines (see www.icmje.org). The authors have stated that no such relationships exist.

## Conflict of Interest

All authors have affirmed they have no conflicts of interest to declare.
