# Supplementary figures and images for: Evaluating the Accuracy of Laryngoscopic View Documentation During Tracheal Intubation in a Pediatric Emergency Department
Source: J Am Coll Emerg Physicians Open. 2025 Jul 24;6(5):100227. doi: 10.1016/j.acepjo.2025.100227 (PMC12312023; doi:10.1016/j.acepjo.2025.100227)

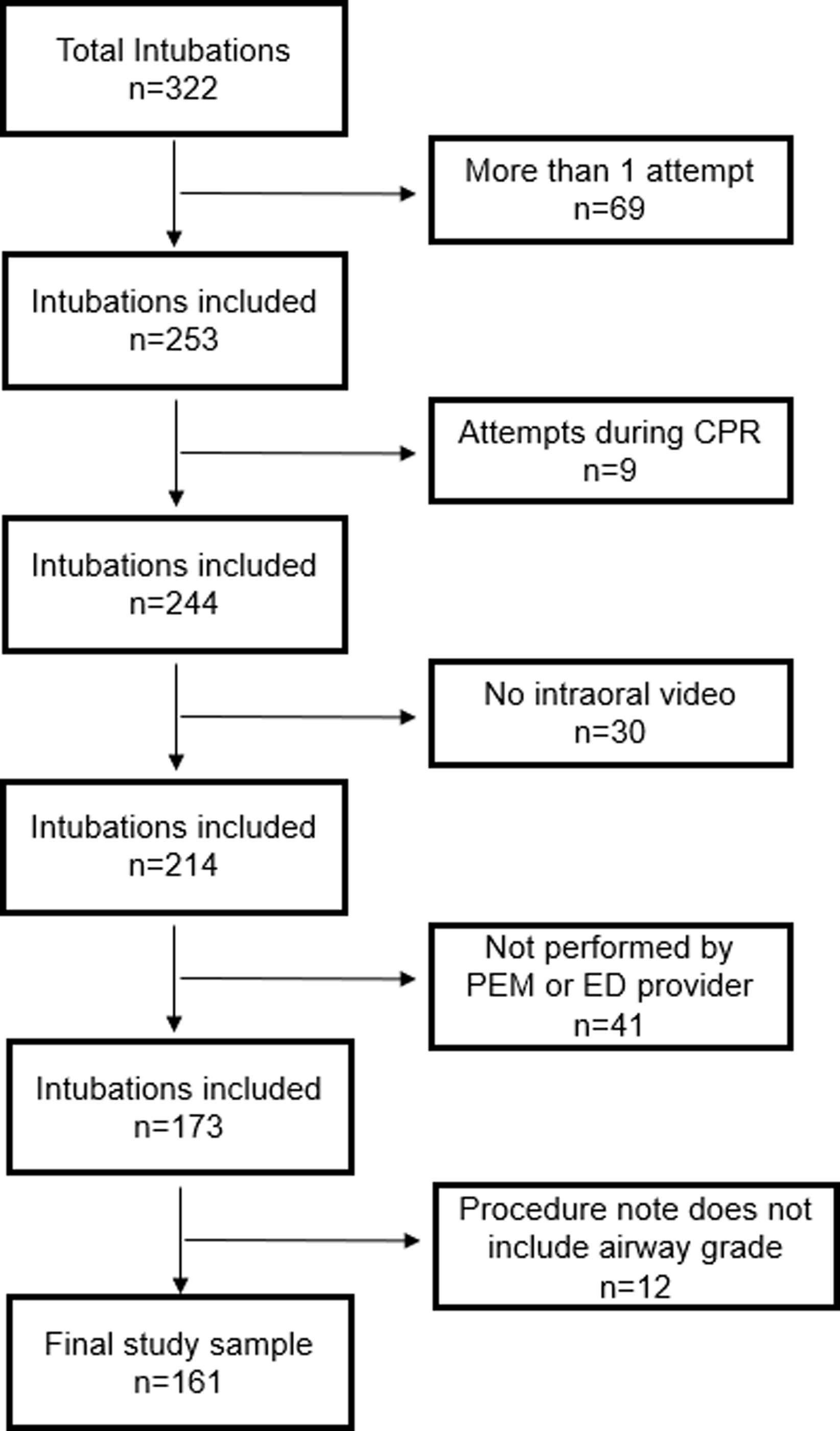

Supplement: Figure S1 [file figs1.jpg]
